# Radical chemistry in polymer science: an overview and recent advances

**DOI:** 10.3762/bjoc.19.116

**Published:** 2023-10-18

**Authors:** Zixiao Wang, Feichen Cui, Yang Sui, Jiajun Yan

**Affiliations:** 1 School of Physical Science and Technology, ShanghaiTech University, 393 Middle Huaxia Rd., Shanghai, 201210, China

**Keywords:** crosslinking, polymer surface modification, post-polymerization modification, radical chemistry, radical polymerization

## Abstract

Radical chemistry is one of the most important methods used in modern polymer science and industry. Over the past century, new knowledge on radical chemistry has both promoted and been generated from the emergence of polymer synthesis and modification techniques. In this review, we discuss radical chemistry in polymer science from four interconnected aspects. We begin with radical polymerization, the most employed technique for industrial production of polymeric materials, and other polymer synthesis involving a radical process. Post-polymerization modification, including polymer crosslinking and polymer surface modification, is the key process that introduces functionality and practicality to polymeric materials. Radical depolymerization, an efficient approach to destroy polymers, finds applications in two distinct fields, semiconductor industry and environmental protection. Polymer chemistry has largely diverged from organic chemistry with the fine division of modern science but polymer chemists constantly acquire new inspirations from organic chemists. Dialogues on radical chemistry between the two communities will deepen the understanding of the two fields and benefit the humanity.

## Introduction

Early last century, with the groundbreaking macromolecular hypothesis by Hermann Staudinger [1], polymer science was born out of organic chemistry. Since then, polymer science has evolved into an important branch of physical science and a fundament of the modern life. Like many other organic methodologies, radical chemistry was applied to polymer science and nowadays, radical chemistry plays a critical role in both the production of a major portion of industrial polymers and the research on novel materials [2]. In this minireview, we discuss several aspects of radical chemistry found in polymer science.

Section 1 focuses on the best-established radical chemistry – radical polymerization, including radical polymerization in nature, conventional radical polymerization, and a new class of radical polymerization, reversible deactivation radical polymerization, which emerged late last century. To continue with the discussion on polymer construction, section 2 explores some emergent polymer synthesis techniques via a radical process but other than a chain-growth mechanism by addition of radical species to vinyl monomers. In section 3, we cover radical chemistry approaches used in post-polymerization modification, including chemical crosslinking of polymers and polymer surface modification. Radicals are powerful tools for post-polymerization processes because of their exceptional reactivity. In contrast to the previous sections, we set the topic of section 4 on the radical degradation of polymers, both in nanofabrication and polymer upcycling.

## Review

### 1 Radical polymerization

Radical polymerization has long been an effective and inexpensive method in the synthesis of polymers since it was invented, making it the most important industrial polymerization technique. Polymers produced by radical polymerization represent a major fraction of all industrial polymers.

#### 1.1 Radical polymerization in nature

**Scheme 1:**
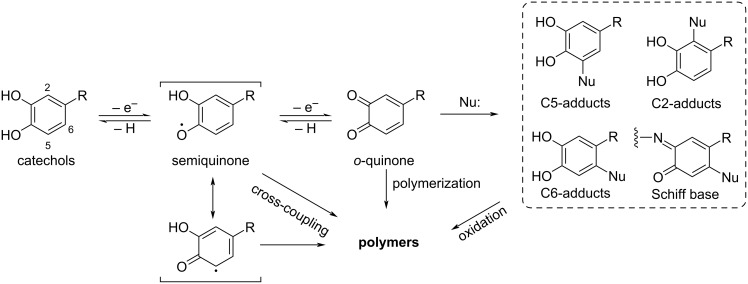
Oxidation of catechol and subsequent cross-linking. Scheme 1 redrawn from [3].

In addition to well-established processes in modern industry, examples of radical polymerization exist in nature. The principle of the two cases in the following text is based on the radical polymerization of catechol derivatives. Catechols are known as easily oxidizable compounds and are prone to undergo oxidation by losing one or two electrons [3]. This way, either semiquinone radicals or *o*-quinones are formed by single or double-electron oxidation, respectively [4]. The semiquinone radicals formed during the oxidation of catechol can undergo a cross-coupling reaction to form polymers (Scheme 1).

**Scheme 2:**
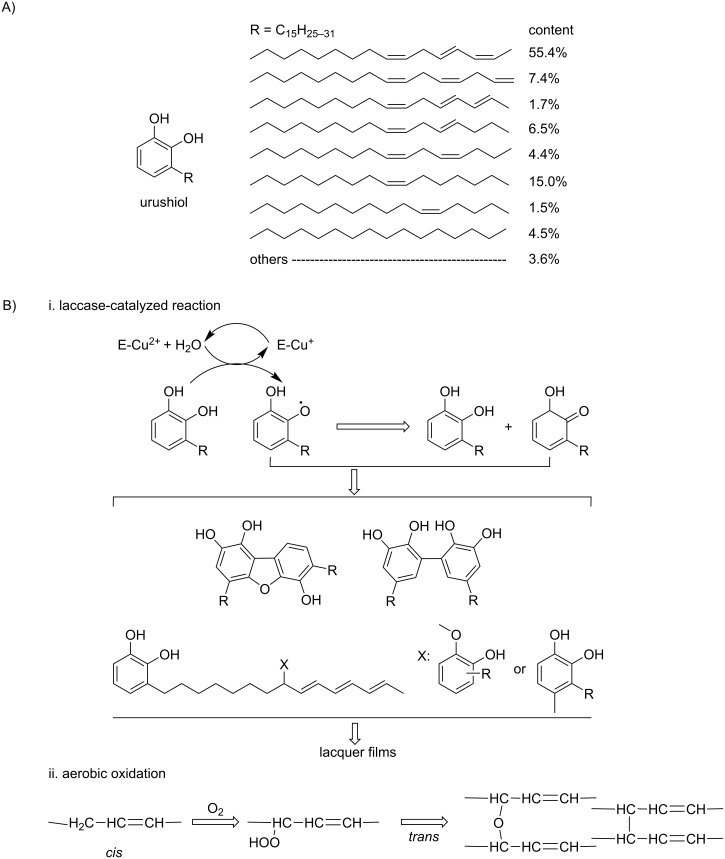
(A) Structure of typical urushiol in Chinese lacquer, and (B) schematic process of laccase-catalyzed oxidation polymerization of Chinese lacquer and autoxidation reaction on the long aliphatic unsaturated side chain. Scheme 2 redrawn from [9].

One example is the radical polymerization of urushiol. The earliest recorded application of natural radical polymerization can be traced back to the manufacture of lacquerwares several thousand years ago [5]. The surface coating of lacquerwares was made up of a sap from a lacquer tree growing in Asia. The lacquer sap obtained from *Rhus vernicifera* lacquer tree mainly consists of urushiol (60–65%), water (20–30%), lacquer polysaccharide (3–7%), water-insoluble glycoprotein (≈1–2%), laccase (≈0.2%), and stellacyanin (≈0.02%) [6–7]. Urushiol is the main active coating-forming ingredient of the resin. A typical urushiol is shown in Scheme 2. In a humid and warm environment, urushiol absorbs oxygen from air and is oxidized to a phenolic oxygen free radical under the action of laccase enzymes [5]. The radical then rearranges to form a semiquinone radical and reacts rapidly with a neighboring urushiol molecule to produce a biphenyl dimer. The dimers further polymerize to form the polymer [8].

**Scheme 3:**
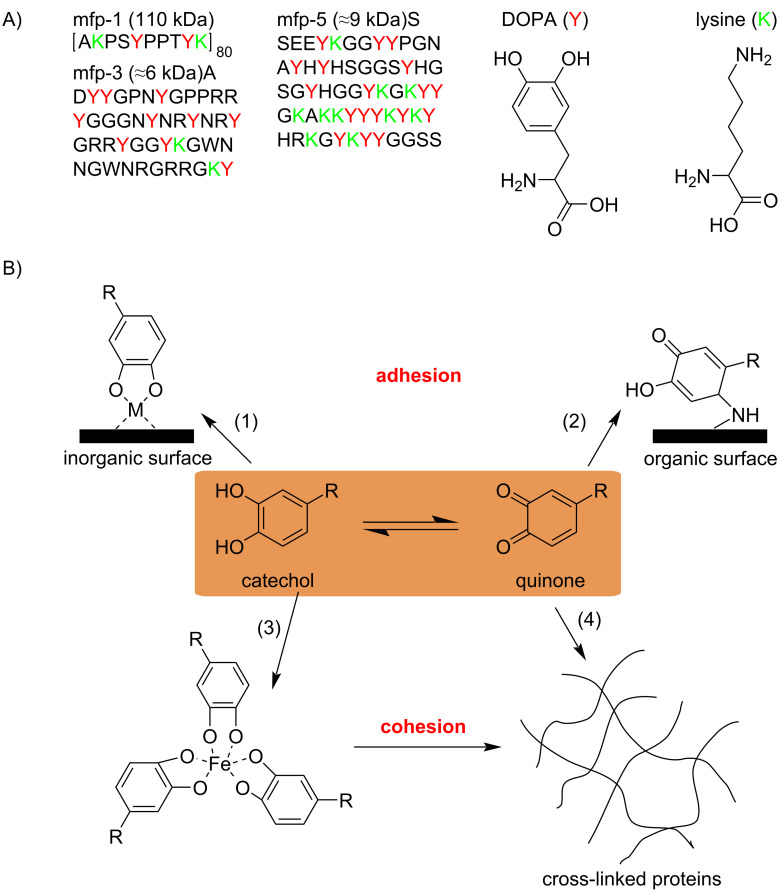
A) Primary amino acid sequence of mfp-1, mfp-3, and mfp-5 (Y: DOPA, K: lysine). B) Scheme showing examples of the adhesive and cohesive properties of catechol-containing proteins. R represents the remainder of the mfp’s. Scheme 3 redrawn from [10].

Radical processes also occur in oceans. The mussel attachment system consists of a bundle of disc-tipped acellular thread called *byssus,* which connect the mussel to the surfaces of substrates [10]. A family of proteins called mussel foot proteins (mfp’s) distribute throughout the whole length of *byssus* while there is an extremely high concentration of mfp’s at the plaque–substrate interface. The mfp's contain up to 27 mol % of DOPA (ʟ-3,4-dihydroxyphenylalanine), which plays a crucial role on mussel adhesion [11]. Although the crucial role of DOPA in mussel adhesion is not fully understood, a prevailing view suggests that DOPA can be oxidized to *o-*quinones at an acidic pH and the quinones react with unoxidized catechols to form *o-*semiquinone radicals afterwards [12]. The semiquinone radicals can help DOPA adhere onto organic surfaces. At a basic pH, the system is cured and mechanically stabilized through the formation of DOPA-metal coordination bonds. The cohesion of the DOPA-metal complex helps mussel adhere onto inorganic surfaces (Scheme 3).

#### 1.2 Conventional radical polymerization

Radical polymerization, which IUPAC defines as ‘A chain polymerization in which the kinetic-chain carriers are radicals’ [13], is the most widely used reaction in polymer industry. As far back as the 1950s, the basic theory and comprehension of radical polymerization was established. In the past decades, radical polymerization was introduced to be an efficient industrial synthesis method to produce numerous chemicals such as low-density polyethylene (LDPE), polystyrene (PS), and poly(vinyl chloride) (PVC) [14]. About half of the industrial polymers manufactured worldwide are produced by radical polymerization [15–16].

**1.2.1 Key features of radical polymerization:** Radical polymerization, which has a classical chain reaction process, usually analyzed kinetically on the assumption of a steady state with respect to the concentration of chain carriers (radicals) [17]. Radical polymerization is a complex mechanism. The basic reactions have been known for quite some time now. They can be simply described by 8 equations (Equations 1–8) as follows. In the equations, I_2_ represents an initiator molecule; M represents a monomer molecule; P*_i_* represents a polymer chain with *i* repeating units; S represents a solvent molecule; and a dot indicates free radical species. These reactions are classified into four elementary steps: initiation, propagation, termination, and transfer. The initiation step includes Equation 1 and Equation 2, when the thermal initiator decomposes into two small-molecule radical species and the first monomer adds to the growing chain to form the first repeating unit. The propagation step is depicted in Equation 3 when monomers take turns to undergo radical addition. The termination step occurs by either disproportionation (radical β-elimination, Equation 4) or biradical coupling (Equation 5). Chain transfer (Equations 6–8) is usually considered as a type of side effect in radical polymerization [18]. It occurs between the growing chain and a transfer agent, which can be the monomer (Equation 6), the solvent (Equation 7), the polymer itself (Equation 8), or a chain-transfer agent intentionally added to tune the molecular weight or to introduce chain-end functionalities. When chain transfer happens, the originally growing chain halts while a new chain launches from the radical species formed from the chain transfer agent. In the case when a growing chain-end radical transfers to its own backbone, i.e., backbiting occurs, a branching point forms as propagation continues in the middle of the backbone.


[1] $${\text{I}}_{2}\overset{k_{\text{d}}}{\to }2{\text{I}}^{•}$$

[2] $${\text{I}}^{•}\text{ + M}\overset{k_{\text{d}}}{\to }{\text{ P}}_{1}^{•}$$

[3] $${\text{P}}_{n\;}^{•}+\text{M}\overset{k_{\text{p}}}{\to }{\text{P}}_{n+1}^{•}$$

[4] $${\text{P}}_{n}^{•}{\text{ + P}}_{m}^{•}\overset{k_{\text{td}}}{\to }{\text{ P}}_{n}^{=}+{\text{ P}}_{m}$$

[5] $${\text{P}}_{n}^{•}{\text{ + P}}_{m}^{•}\;\overset{k_{\text{tc}}}{\to }{\text{ P}}_{n\text{+}m}$$

[6] $${\text{P}}_{n}^{•}\text{ + M }\overset{k_{\text{tr}}^{\text{mon}}}{\to }{\text{ P}}_{n}{\text{ + M}}^{•}$$

[7] $${\text{P}}_{n}^{•}\text{ + S }\overset{k_{\text{tr}}^{\text{sol}}}{\to }{\text{ P}}_{n}{\text{ + S}}^{•}$$

[8] $${\text{P}}_{n}^{•}{\text{ + P}}_{m}\;\overset{k_{\text{tr}}^{\text{pol}}}{\to }{\text{ P}}_{n}{\text{ + P}}_{m}^{•}$$

Radical polymerization is applicable to a large number of vinylic monomers and is tolerant toward many solvents, functional groups, and impurities common in industrial systems, which makes it an ideal choice for industrial production [19]. Vinylic monomers should be thermodynamically and kinetically polymerizable. The former requires a sufficiently negative free energy of polymerization and the latter an adequate reactivity of the monomer, stability of the derived free radical, and a low proportion of side reactions.

A slow rate of chain initiation, a fast rate of chain propagation, and a rapid rate of chain termination are key features of conventional radical polymerization. Most free radicals have an extremely short lifetime due to a diffusion-controlled termination process between two free radicals. The inevitable termination between radicals makes the synthesis of well-defined polymers and co-polymers very difficult. In addition, polymers obtained from conventional radical polymerization are commonly linear polymers, though transfer to polymer may induce branching.

**1.2.2 Radical polymerization in modern industry:** In the commercial production of high-molecular-weight polymers, radical polymerization is a widely used method. Its main advantages are (i) the universality to a wide range of monomers such as (meth)acrylates, (meth)acrylamides, dienes, vinyl ethers/esters, etc.; (ii) tolerance to unprotected functionalities in monomers and the solvent including –OH, –COOH, –SO_3_H, etc.; (iii) different reaction conditions, including bulk, solution, emulsion, and suspension; and (iv) inexpensive and facile set-ups compared to other polymerization techniques [20].

There are four common industrial methods of radical polymerization [2] as shown in Table 1.

**Table 1:** Common ways of radical polymerization in industry.

method	contains	where polymerization happens

bulk polymerization	monomer, initiator	in bulk
solution polymerization	monomer, initiator, solvent	in solution
suspension polymerization	hydrophobic monomer, hydrophilic initiator, water, suspending agents	in monomer droplet
emulsion polymerization	hydrophobic monomer, hydrophilic initiator, water, surfactants	in latex/colloid particles

The only components of a bulk polymerization mixture are monomers, the initiator, and optionally, a chain-transfer agent [21]. Products obtained from bulk polymerization have high optical clarity and are usually very pure [2]. The mechanism and equipment are relatively simple for a large-scale production in a short time. However, heat and mass transfer become difficult as the viscosity of the reaction mixture increases. This may lead to autoacceleration, also known as the Trommsdorff–Norrish effect, or even a violent explosive polymerization. At the same time, heat acquisition may cause a broad molecular weight distribution.

Solution polymerization can effectively mitigate problems of bulk polymerization. The use of a solvent can lower the viscosity of the polymerization system, leading to better mass and heat transfer. Good heat transfer can reduce the Trommsdorff–Norrish effect [22]. Meanwhile, the inhibited termination reactions cause a significant increase in the overall yield.

Polyacrylonitrile (PAN), polyacrylic acid (PAA), and polyacrylamide (PAM), for instance, are obtained by solution polymerization in the polymer industry [23–25]. In addition to the reduced reaction rate due to lower monomer and initiator concentrations, one of the major disadvantages of solution polymerization is that it is difficult to completely rule out chain transfer to the solvent. Therefore, obtaining very high molecular weight product through solution polymerization is tough.

Suspension polymerization is a heterogeneous process and requires the use of a mechanical agitation to mix monomers and dissolved initiators in the liquid phase during the process. A suspending agent, e.g., polyvinyl alcohol (PVA), is added to the system to prevent coalescence. The viscosity in suspension polymerization is low throughout the process which brings good heat transfer and temperature control, and therefore well-defined and high-molecular-weight polymers. PVC, PS, and poly(methyl methacrylate) (PMMA) are industrially produced through suspension polymerization [2]. Nonetheless, the Trommsdorff–Norrish effect exists in suspension polymerization processes, and the residual suspending agent becomes an impurity.

Emulsion polymerization is also a widely-used method in radical polymerization. It is applied to produce several commercially important polymers such as acrylic rubber, nitrile rubber, and polytetrafluoroethylene (PTFE). Polymerization happens in latex or colloid particles that are formed under the action of surfactants, which are also called emulsifiers within the first few minutes [26]. Emulsifiers such as sodium lauryl sulfate, sodium or potassium salts of fatty acids (soaps), salts of alkylbenzene sulfonates, and *O*-polyoxyethyleneated long-chain alcohols are used to change the two incompatible water phase and oil phase into an emulsion phase. The simultaneous presence of a hydrophobic head and a hydrophilic tail on emulsifiers provides the ability to combine water and oil phase into an emulsion. In emulsion polymerization, high molecular weights can be achieved at fast polymerization rates, because both the rate of polymerization and the molecular weight depends on the number of particles. A small latex particle only rooms a single propagating radical at a time. Thus, the chain keeps growing until another radical enters to terminate it. Due to the enormous number of particles, the overall radical concentration in the latex is greatly higher than in a typical bulk polymerization. Meanwhile, the polymerization rate is higher in emulsion polymerization compared to bulk or suspension polymerization. Radicals are divided in different particles also allows for longer lifetimes, which results in a higher degree of polymerization. As the frequency of radical entry decreases with the particle number at a certain initiator concentration, the rate of polymerization and molecular weight can be boosted by raising the number of particles, e.g. by tuning the monomer to surfactant ratio.

The final product can be used as is and does not generally need to be altered or processed. Drawbacks of emulsion polymerization include residual surfactants, significant chain transfer to polymer, and difficulty to dry polymers.

#### 1.3 Reversible deactivation radical polymerization

A key drawback of conventional radical polymerization is that a limited control of molecular weights and architectures can be achieved due to the slow initiation and rapid termination. In 1956, Szwarc coined the term “living polymerization” in an anionic system [27]. Since then, polymer chemists have been in pursuit for a comparable “living radical polymerization”. Despite the fact that radical polymerization is never as “living” as the anionic counterpart, RDRP as per the IUPAC definition, or more commonly named controlled radical polymerization (CRP) has made a booming progress and attracted great attention in the past three decades [28].

**1.3.1 Deactivation by reversible coupling:** In 1982, Otsu and Yoshida [29] successfully polymerized styrene and MMA using dithiocarbamate compounds, and in 1986, Solomon et al. [30] published a patent entitled "Polymerization Processes and Polymers Produced Thereby", which led to the successful nitroxide-mediated polymerization (NMP). In 1993, Georges et al. used benzoyl peroxide (BPO) as the initiator and 2,2,6,6-tetramethyl-1-piperidinyloxyl (TEMPO) as the control agent. It was called a bicomponent initiating system containing both stable free nitroxide and a conventional thermal initiator. Polystyrene of different molecular weights was obtained with low *M*_w_/*M*_n_ and active chain ends [31].

**Scheme 4:**
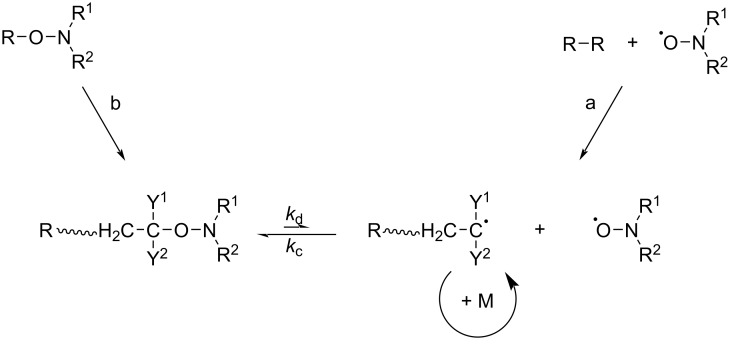
Activation–deactivation equilibrium in nitroxide-mediated polymerizations. Bicomponent initiating system (a) and unicomponent initiating system (b). Scheme 4 redrawn from [35].

Although a bicomponent initiating system is economical and practical, the traditional initiators have many problems such as the poor initiation efficiency. It is difficult to control the molecular weight and polymerization rate precisely. In order to solve these problems, Hawker et al. [32–34] proposed the concept of the unimolecular initiation system. In this system, an alkoxyamine compound is used instead of the original nitroxide radical/initiator combination. These unimolecular initiators can decompose to produce a stoichiometric pair of the primary initiating radical and a nitroxide radical, thus combining the roles of a conventional initiator and a control agent. The mechanism is shown in Scheme 4 [35].

Due to the steric effect of TEMPO, the dissociation rate constant, *k*_d_, of the corresponding alkoxyamine is very low and it tends to undergo β-elimination in acrylic systems. Thus, TEMPO is only suitable for the polymerization of styrenic monomers at a high temperature and for long time [36]. Functionalized TEMPO was therefore developed for the polymerization of other monomers, such as acrylates, under milder conditions [37–39]. Grimaldi et al. [40] achieved NMP of styrene and *n*-butyl acrylate using SG1-type nitroxide radical (*N*-*tert*-butyl-*N*-(1-diethylphosphono-2,2-dimethylpropyl)nitroxide). Compared with TEMPO, SG1 was considered that it initiated the truly “living”/controlled polymerization at that time and the rate of propagation was much faster than with TEMPO under the same conditions.

**Scheme 5:**
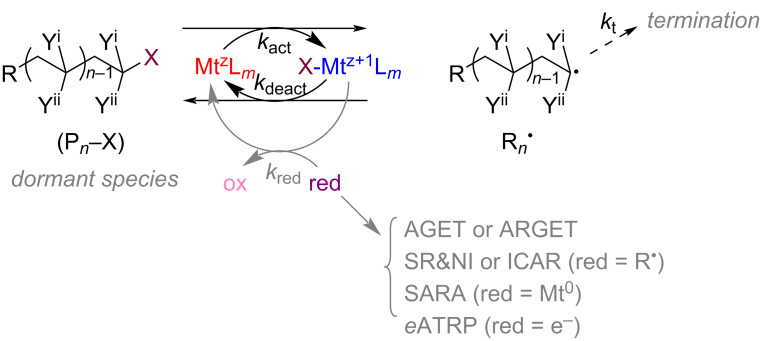
Mechanism of a transition metal complex-mediated ATRP. Scheme 5 redrawn from [14].

**1.3.2 Deactivation by atom transfer:** Atom transfer radical polymerization (ATRP) was independently reported by the teams of Matyjaszewski [41] and Sawamoto [42] in 1995. The efficient conduct of ATRP relies on the establishment of a reversible activation/deactivation equilibrium reaction between an alkyl halide or halide-like initiator (RX) and a radical species (R^·^) [43]. During the activation process, the organohalides quickly lose their terminal halogen atoms in the presence of the liganded low-valent metal complex (activator, Mt^z^/L, typically Cu^I^/L) to form the active radical species (R^·^), which in turn initiates polymerization to form the active polymer chain species (P*_n_*^·^). On the other hand, the termination reactions always present in the system causing the liganded high-valent metal complex (deactivator, X–Mt^z+1^/L, typically X–Cu^II^/L) to accumulate. When the accumulation reaches a certain level, the deactivator interacts with the active radical chain species (P*_n_*^·^), so that the radical chain species gets into the dormant state (P*_n_*X) via a halogen atom transfer process. This is the deactivation process. Activation and deactivation reactions are always present throughout the process, and the rate of deactivation must be sufficiently high in order to maintain a low radical concentration to effectively inhibit the termination [44]. The mechanism of ATRP is shown in Scheme 5 [14].

Compared with the earlier ATRP techniques (normal ATRP [41], reverse ATRP [45], SR&NI ATRP [46], and AGET ATRP [47]), the recently proposed ATRP techniques (ICAR ATRP [48], ARGET ATRP [49], SARA ATRP [50], *e*ATRP [51–52], photoATRP [53–54], and ultrasonic ATRP [55]) require a much lower catalyst dosage, even down to 10 ppm [56], which to some extent, solves the problem of metal impurities. Meanwhile, the presence of external stimuli in *e*ATRP, photoATRP, and ultrasonic ATRP, allows spatial and temporal control over the polymerization [57]. Hawker et al. proposed a metal-free ATRP in 2014 using an organic photoredox catalyst mediated by light to overcome the challenge of metal contamination in the precipitated polymers [58]. After the ATRP reaction, a reactive chain end retains as a stable alkyl halide moiety. Therefore, ATRP is particularly suitable for the synthesis of polymers with complex architectures [59–60].

**1.3.3 Deactivation by degenerative transfer:** Reversible addition-fragmentation chain transfer (RAFT) polymerization is one of the most well-established RDRP technique. It was first proposed in 1998 by Commonwealth Scientific and Industrial Research Organization (CSIRO) researchers Chiefari et al. [61]. Due to the presence of chain-transfer agents (CTAs), such as thiocarbonylthio compounds, in RAFT polymerizations, the chain propagating radical species can add to CTAs to form intermediate radical species. RAFT can rely on reversible chain-transfer reactions between the propagating radical species and the dormant chains to achieve controlled polymerization of the monomers, allowing all polymer chains to grow nearly simultaneously.

**Scheme 6:**
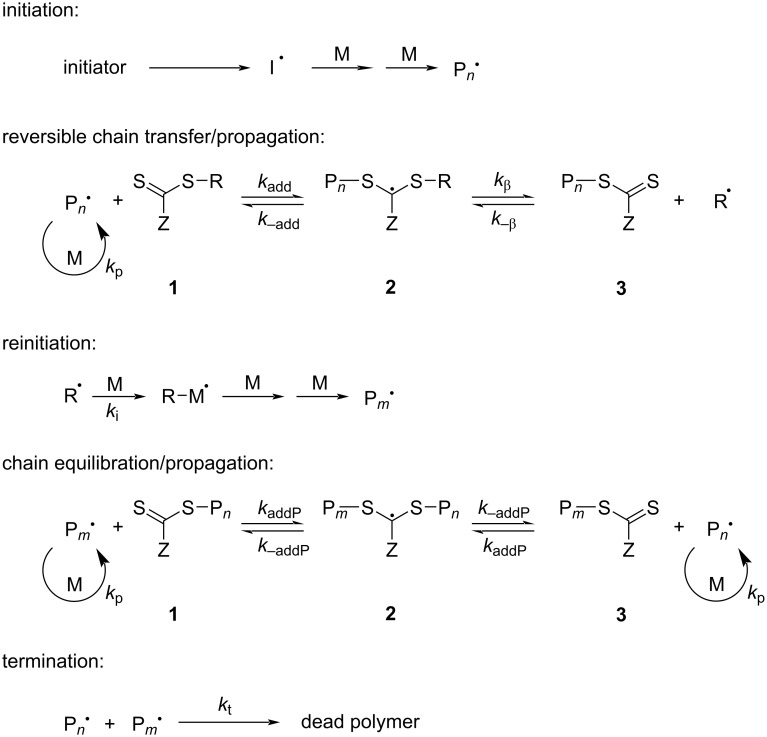
Mechanism of RAFT polymerization. Scheme 6 redrawn from [68].

The product of RAFT polymerization has a preserved thiocarbonylthio chain end. The polymerization reaction can be continued by adding more monomers. Therefore, RAFT is often used to perform chain expansion reactions or to synthesize functionalized multi-block copolymers [62–64]. Boyer and co-workers developed a photocatalytically mediated RAFT polymerization, PET-RAFT, which removes the requirement for conventional radical initiators. The reaction is oxygen tolerant and can be carried out in a milder environment [65–66]. Pan and co-workers recently further advanced the RAFT techniques by allowing them to be fueled by oxygen [67]. The mechanism of a RAFT polymerization is shown in Scheme 6 [68].

**Scheme 7:**
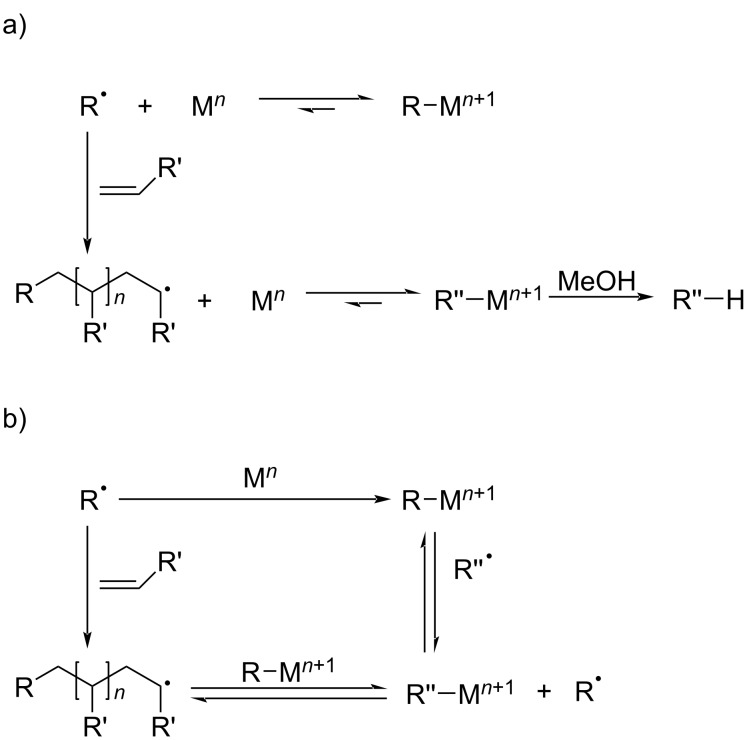
Degenerative transfer (a) and reversible termination (b) mechanism of OMRP. Scheme 7 redrawn from [70].

Organometallic-mediated radical polymerization (OMRP) is also a commonly used polymerization method, which uses transition-metal complexes such as titanium and vanadium for coordination polymerization [69]. However, due to the high cost of these complexes and their post-processing, OMRP is not widely used. The chain termination reaction of OMRP is considered to have two mechanisms, degenerative transfer and reversible termination, which are comparable to RAFT and NMP, respectively (Scheme 7) [70].

**Scheme 8:**
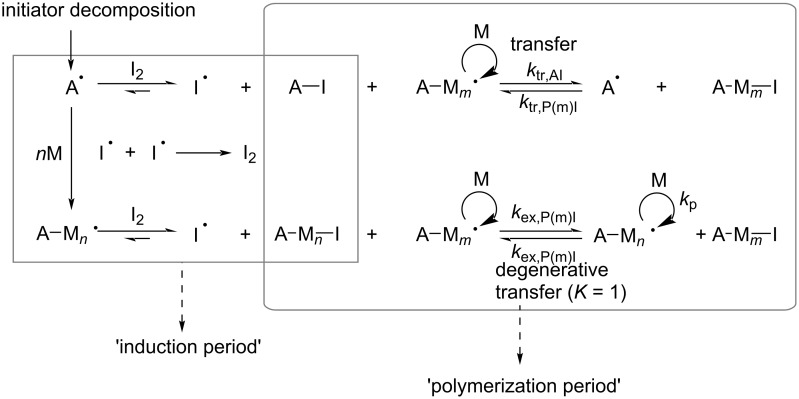
Simplified mechanism of a RITP. Scheme 8 redrawn from [21].

Iodine transfer polymerization (ITP) is also a commonly used degenerate chain-transfer method. Its origin can be traced back to the 1970s [71] and it is mostly used for the polymerization of fluorinated olefins. However, the C–I bond of iodoalkyl compounds used as chain-transfer agents is weak and unstable during storage [21]. Therefore, Lacroix-Desmazes et al. [72] used iodine molecules to synthesize iodine chain transfer agents in situ, a process known as reverse iodine transfer polymerization (RITP), which is similar to reverse ATRP. The mechanism of the RITP is shown in Scheme 8.

RDRP is applicable to a wide range of monomers and the reaction conditions become milder and more versatile with emerging techniques, such as oxygen tolerance or even oxygen initiation [73]. Compared with the conventional radical polymerization, RDRP has shown fascinating advantage in complex polymer polymerization. However, RDRP is currently less applied in industry due to cost and process obstacles. It is expected that future technical innovation will allow RDRP to be more widely employed.

### 2 Other polymerization techniques involving radical chemistry

As discussed in section 1, chain-growth polymerization via radical addition to vinyl monomers is the most broadly applied polymerization technique. However, radical chemistry is used in other polymerization systems. In this section, we cover these techniques excluded from our previous discussion.

#### 2.1 Oxidative synthesis of conductive polymers

**Scheme 9:**
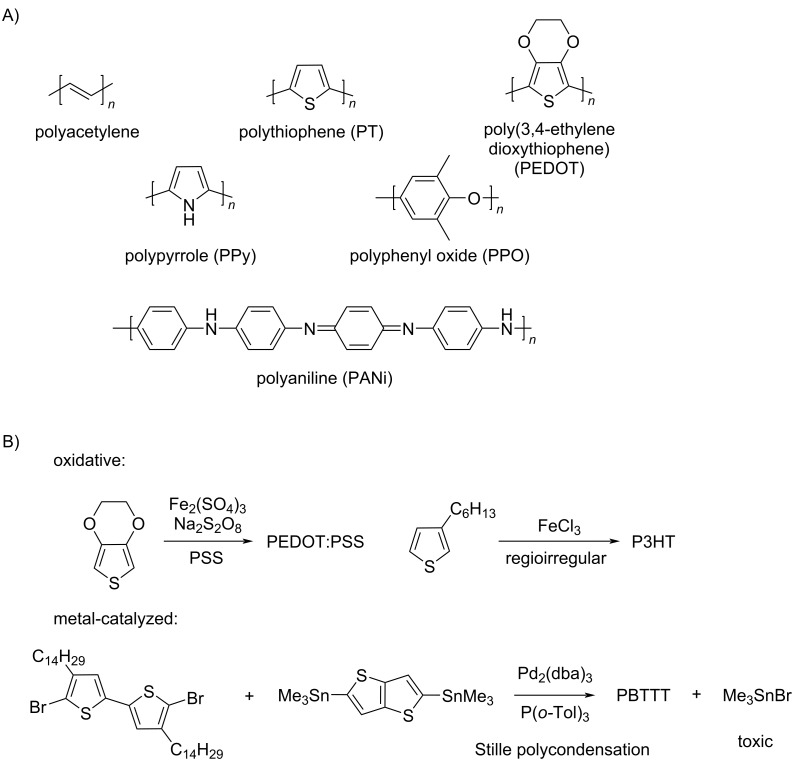
(A) Structures of π-conjugated conductive polymers. (B) Examples of conductive polymer synthesis via chain-growth polymerization. Scheme 9 redrawn from [76].

The breaking accomplishments of Shirakawa, MacDiarmid, and Heeger have changed our view of organic polymers, from insulating polymers to electrically (semi)conducting materials [74]. In 2000, they received the Nobel Prize in Chemistry. Typical conductive polymer structures have π-conjugation (Scheme 9A) [75]. They can be synthesized by various methods such as electrochemical and chemical methods. Oxidative polymerization and chain-growth polymerization are also good ways to produce conductive polymers (Scheme 9B) [76].

**Scheme 10:**
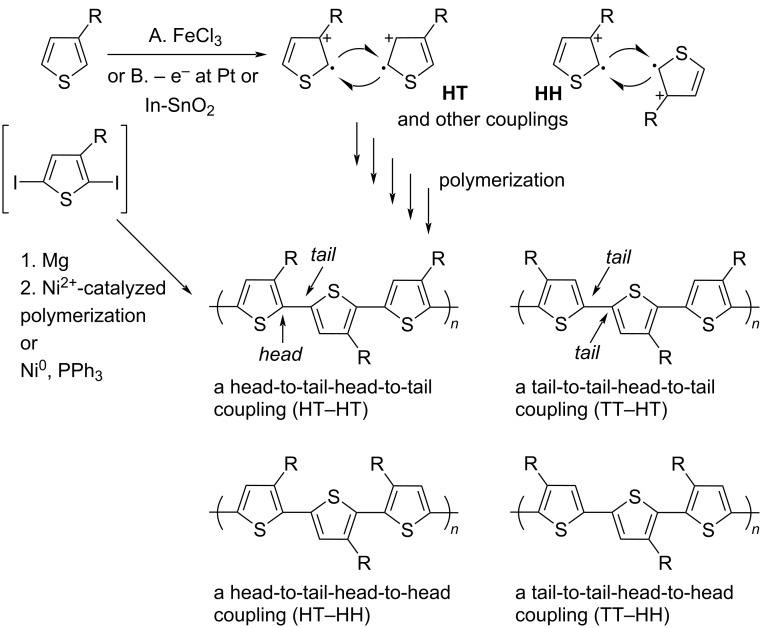
Possible regiochemical couplings in PATs. Scheme 10 redrawn from [79].

Nowadays, most conductive polymers are prepared via metal-catalyzed cross-coupling reactions [77]. However, radical polymerization is also an effective way to synthesize conductive polymers at a relatively low cost. Niemi et al. [78] used FeCl_3_ as catalyst to produce radicals at the 2- and 5-positions of thiophene and synthesized four types of poly(3-alkylthiophene)s (PATs) with different linking ways (Scheme 10).

#### 2.2 Polymerization by thiol–ene chemistry

The thiol–ene reaction (also called alkene hydrothiolation) is the anti-Markovnikov addition of a thiol to a C–C double bond and was first reported in 1905 [80]. It is considered as a click chemistry reaction due to its high yield, stereoselectivity, rate, and thermodynamic driving force.

**Scheme 11:**
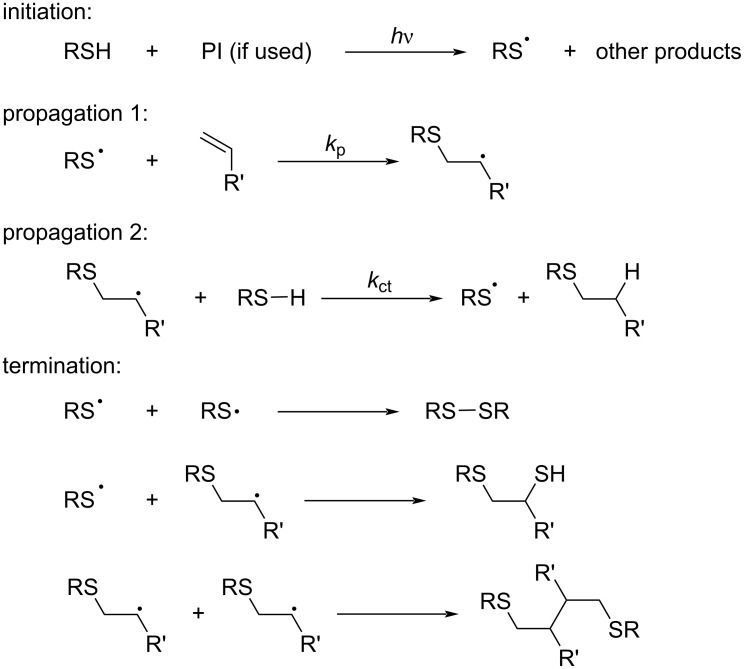
General thiol-ene photopolymerization process. Scheme 11 redrawn from [81].

Generally, the thiol–ene reaction is conducted under radical conditions, often photochemically induced [81]. In a typical thiol–ene system, the polymerization undergoes a free-radical chain mechanism, involving an initiation step from a thiol group via radical transfer or homolysis (Scheme 11, initiation), radical addition of the thiyl radical to the ene functionality (propagation 1), transfer from the carbon-centered radical to another thiol group (propagation 2), and biradical termination between either carbon-centered or thiyl radicals (termination).

Polymerization by thiol–ene coupling is a step-growth polymerization, which means it can produce polymers with no theoretical upper-limited molecular weight. The simple setup, mild conditions, absence of unfavored byproducts, orthogonality with other reactions, and high yields (nearly full conversion) [82] made thiol–ene polymerizations an ideal way to produce high-molecular-weight cross-linked polymers, optical polymers, biomacromolecules, and materials used in additive manufacturing. It is also compatible to a photopolymerization process. For example, it can be applied to the photo-3D-printing of silicone resin [83]. The refractive index is one of the most important optical properties and researchers have invested plenty of effort to develop high refractive-index polymers. A common approach is to incorporate atoms or groups with high polarizability and sulfur is a typical constituent with high molar refractivity and is widely used in optical polymers. For example, Bhagat et al. [84] produced polymers with high cross-linking density and refractive index from tetravinylsilane, ethanedithiol, and benzenedithiol. Polymers made by thiol–ene polymerization usually have well-ordered molecular networks. This character gives thiol–ene polymers highly tunable mechanical response hence it shows great application potential in additive manufacturing. Cook et al. [85] presented the first report of volumetric additive manufacturing-printed thiol–ene resins and showed the potential of the thiol–ene system.

In addition to thiol–ene chemistry, radical hydrosilylation was also used to prepare linear, branched, or cross-linked polymers via a step-growth mechanism (cf. section 3.2) [86].

#### 2.3 Metal-free ring opening metathesis polymerization (MF-ROMP)

**Scheme 12:**
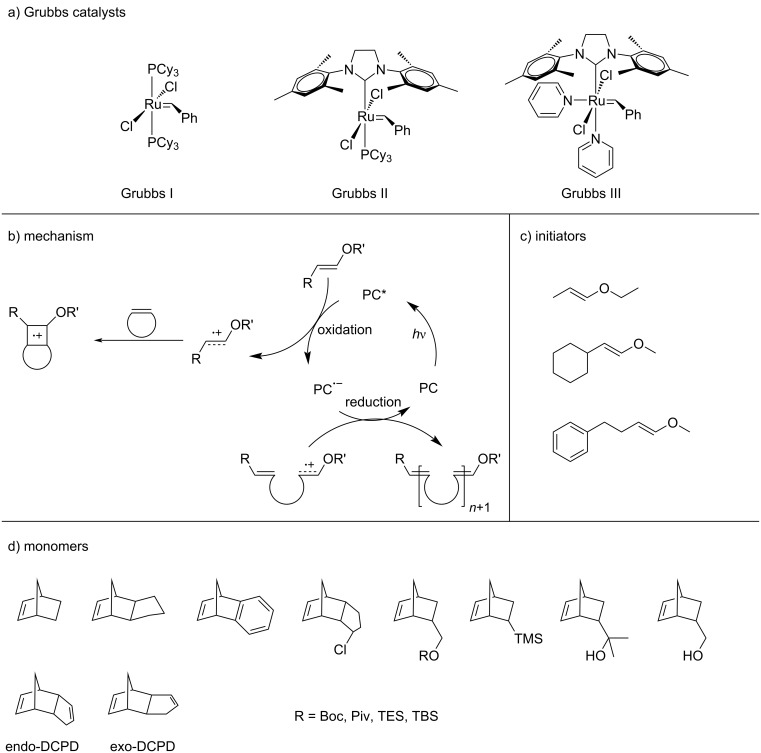
(a) Three generations of Grubbs catalysts. (b) Proposed mechanism for photo-ROMP via a reductive quenching pathway and (c, d) chemical structures of the (c) initiators and (d) monomers used in photo-ROMP. Scheme 12 redrawn from [89].

ROMP is a powerful and broadly applicable technique for synthesizing polymers. Traditional ROMP systems are initiated by transition-metal complexes and Ru-based alkylidene complexes, which are also known as Grubbs catalysts (Scheme 12A), are the most popular ones [87]. However, Ru-based catalysts are expensive making them less attractive for industrial applications. Living ROMP is commonly terminated by adding a special chemical which can remove the transition metal from the chain end and deactivate it from propagation. However, removing this residue from the product by traditional chromatographic methods can be a challenging task and limits the application of ROMP-produced polymers in biomedical and microelectronic fields [88]. To avoid such drawbacks, the development of a metal-free (MF) procedures is necessary.

**Scheme 13:**
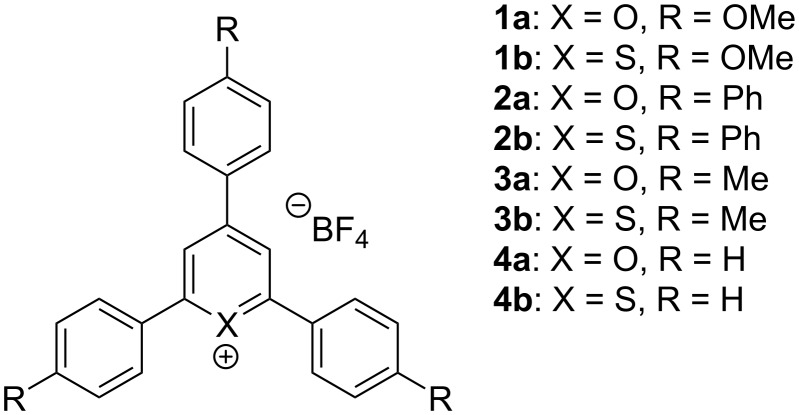
Pyrylium and thiopyrylium salts studied by Boydston et al. Scheme 13 redrawn from [91].

MF-ROMP, also termed photo-ROMP, is a novel technique to polymerize cyclic olefins. It begins with the reductive quenching of an photoexcited photocatalyst (PC) at an enol ether initiator to produce a radical cation carrier [90]. Then, the carrier undergoes cyclic addition with a cyclic olefin monomer to generate a cyclobutene radical cation intermediate. The thermodynamically instable intermediate subsequently forms the propagating radical cation species via a ring-opening process. The reduced PC^•−^ terminates the catalytic loop by reducing the propagating species to provide a polymer chain (Scheme 12B). Boydston and co-workers [91], systematically studied various pyrylium and thiopyrylium PCs (Scheme 13). It is necessary for these PCs having a high excited-state redox potential to oxidize the enol ether initiators. A range of enol ether initiators that has been successfully applied in metal-free ROMP are shown in Scheme 12C.

Meanwhile, metal-free ROMP is applied to monomers like functionalized norbornenes and dicyclopentadienes (DCPD) (Scheme 12D) to synthesize polymers and block copolymers [88,92–93].

### 3 Post-polymerization radical chemistry

Post-polymerization modification is a chemical process that introduces functionalities to backbones or side-groups of pre-synthesized polymers [94–95]. It typically takes place in polymer solutions. On the other hand, surface modification of polymers is a special case of post-polymerization on polymeric solids. Radical chemistry is overwhelmingly more common in the latter because there are other more selective and efficient solution chemistry methods for post-polymerization modification, such as nucleophilic substitution [94,96]. In this section, we discuss the radical chemistry used in both processes.

#### 3.1 Post-polymerization modification

**Scheme 14:**
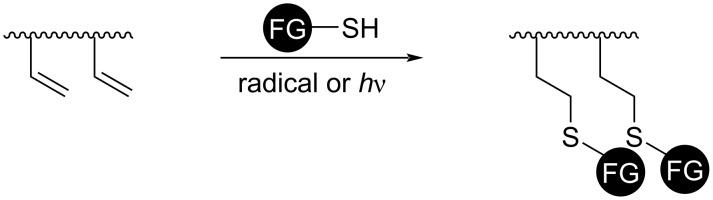
A general illustration of post-polymerization modification by thiol–ene chemistry.

Radical addition is a popular technique for post-polymerization modification of double-bond-containing polymers (Scheme 14). Thiol–ene and thiol–yne “click chemistry” are highly efficient radical processes well-adopted in synthetic chemistry, material fabrication, and chemical biology (cf. section 2.2) [97–98]. The S^·^ radical is typically generated using a thermal initiator or a photochemical process [99–100]. 1,3-Diene polymers are most commonly modified via thiol–ene chemistry through the pendant vinyl after the polymerization [101] and this technique can be traced back to 1948 [102]. The excellent temporal and spatial control of the available photochemical approach makes the technique especially viable for non-solution processes [103]. When a multifunctional thiol is used with diene-functionalized polymers, the approach becomes suitable for chemical crosslinking [103–104], vide infra. It has been used to cure a liquid isoprene polymer in precise digital light processing 3D printing [105]. Recently, Kanbayashi et al. reported that thiol–ene chemistry would not cause racemization of an asymmetric center linked to a pendant vinyl group, which can be particularly valuable for functionalization of optically active polymers [106]. Theato and co-workers introduced vinyl/alkyne-bearing poly(vinyl ether)s [107], poly(vinylcyclopropanes) [108], and poly(allyl 2-ylideneacetate) [109] as promising new platforms compatible to thiol–ene chemistry. Atom transfer radical addition (ATRA) is another process that usually qualifies for a definition of “click chemistry” [44]. A similar radical addition to vinyl groups takes place in ATRA despite the halogen atom transfer is mediated by a metal complex. Post-polymerization modification by ATRA was pioneered by Jérôme and co-workers [110–111]. In 2014, Xu et al. demonstrated that it can be extended to a milder photochemical process as well [112].

**Scheme 15:**
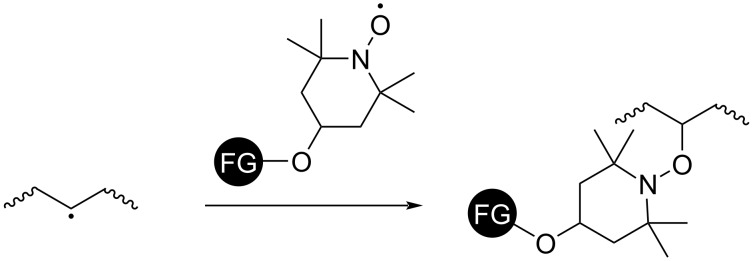
Introduction of functionalities by nitroxide radical coupling of HO-TEMPO derivatives.

Radical coupling may also be used to introduce functional groups into polymer backbones. In this context, rapid radical trapping with stable nitroxide radicals is an efficient way [113]. However, this technique requires radical generation on the polymer backbone. A typical approach involves hydrogen abstraction by organic oxidants such as oxygen radicals from peroxide initiators [114], which is similar to the radical crosslinking process, vide infra. Radicals may also be generated thermally, through photoinduction, or by ATRP initiators incorporated in the polymer backbones [115–117]. TEMPO and its derivatives have a long history of application as radical trapping agents. Commercially available HO-TEMPO is a particularly useful platform for post-polymerization modification via radical coupling because of the chemical versatility of the hydroxy moiety (Scheme 15). Site-selective radical C–H activation has been proven to be a useful tool to functionalize relatively inert polymer backbones and upcycling of polymer waste (cf. section 4) [118–119]. Radical chain-end modification as a highly specific type of post-polymerization modification introduces or removes functionalities at polymer chain ends [120].

#### 3.2 Chemical crosslinking of polymers

Chemical crosslinking is a suitable approach to increase chemical resistance [121], mechanical strength [122], and other properties [123] of polymers. In 1830s, Charles Goodyear invented vulcanized rubber. By heating natural rubber with lead oxide and sulfur, the temperature-sensitive rubber became a more stable material, even at high and low temperatures, while keeping the elasticity, plasticity, insulation, and other excellent characteristics [124]. During the vulcanization of natural rubber, elemental sulfur was heated to form sulfur radicals which then react with natural rubber crosslinking two independent polymer molecules [125]. This is a typical example of polymer crosslinking by a radical mechanism.

**Scheme 16:**
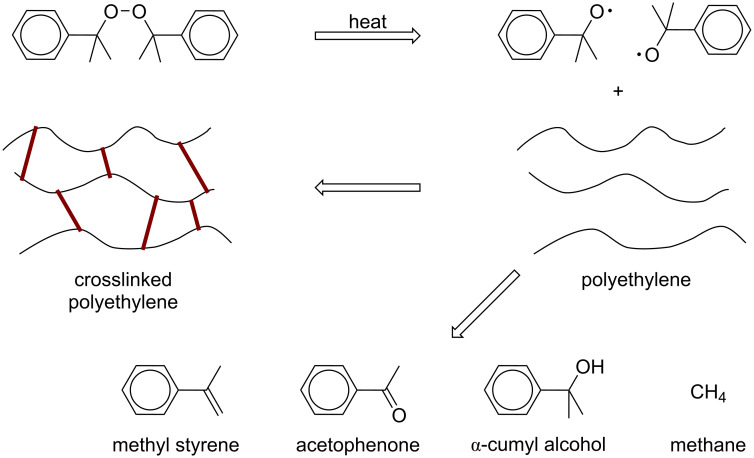
Chemical reaction process scheme of DCP-induced crosslinking of LDPE. Scheme 16 redrawn from [126].

As the traditional vulcanization process, an initiator is needed to start the radical crosslinking. Besides sulfur, peroxides such as di-*tert*-butylcumyl peroxide (BCUP) and dicumyl peroxide (DCP) are often used in radical crosslinking. Free radicals are generated at the peroxides’ decomposition temperature and attack the polymer chains to achieve crosslinking (Scheme 16). In dry crosslinking processing of crosslinked polyethylene (XLPE) used in power delivery system, a blend of DCP in low-density polyethylene (LDPE) is extruded at its melting point. In comparison to LDPE, the operational temperature and the short-circuit permissible temperature of XLPE cables are increased from 70 °C to 90 °C and 150 °C to 230 °C, respectively. Besides that, XLPE shows a more rubber-like behavior [126]. As the peroxide crosslinking process is industrially important, multiple kinetic models have been established to understand the reaction between polymers, peroxides, and monomers [127–129].

**Scheme 17:**
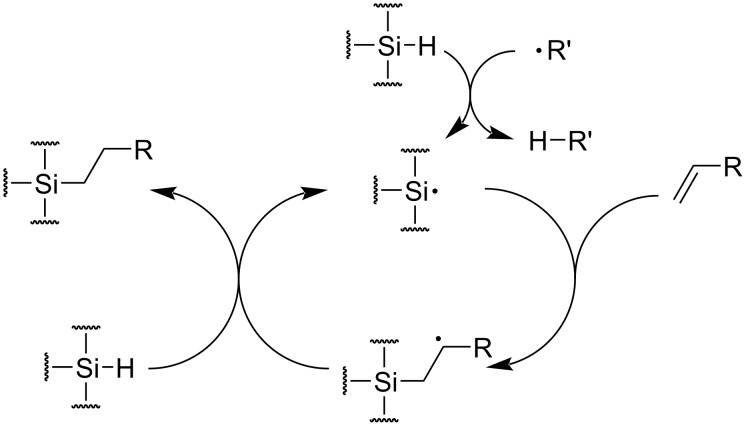
A probable mechanism of radical-induced hydrosilylation.

Polysiloxanes are another class of crosslinkable polymers. Modern silicone industry typically uses Pt-catalyzed hydrosilylation to crosslink multi-vinyl polysiloxane with silicon hydride compounds to manufacture silicone rubbers [130]. However, hydrosilylation may also be achieved via a radical mechanism (Scheme 17). In comparison to the Pt-catalyzed system, the radical-induced hydrosilylation has a lower cost, better tolerance to coordinating functionalities, and yields products without metal residues, but its efficiency is inferior to transition-metal catalyzed methods. Silicone rubber was prepared in such a process [131]. Pan and co-workers recently reported a photoredox hydrosilylation process compatible to both electron-sufficient and -deficient vinyl species [86], and applicable to both post-polymerization modification and crosslinking of polymers bearing pendant vinyl groups [132], demonstrating a promising new orientation of radical hydrosilylation. It is noteworthy, that since the 1940s, polysiloxanes were crosslinked via hydrogen abstraction from Si–CH_3_ and a radical coupling mechanism like polyolefins, vide supra [133].

Irradiation can also lead to the crosslinking of polymers. Polymeric materials may become brittle or colored after being exposed to sunlight for a long time, which was called 'photo-ageing' [134]. In fact, the light sensitivity of many polymers results from some impurities or additives remaining in polymer materials, which can form radical species through irradiation. This photooxidation process can lead to the generation of some small molecules or chain scissoring. At the same time, the photooxidation process can also result in crosslinking of polymer backbones [135]. Bousquet and Fouassier [135] investigated the photooxidation and crosslinking of photosensitized elastomers. Samples of an EPDM (ethylene-propenebutadiene) terpolymer were prepared with different additives. Observable crosslinking products were obtained through irradiation of different wavelengths. Besides the side reactions induced during photoageing, rational photocrosslinking of polymers is also feasible in the presence of photoinitiators or photoresponsive moieties [136–138]. Sophisticatedly designed photocrosslinking of polymers finds broad applications in modern 3D printing/additive manufacturing [139–142].

Radical chemistry has been demonstrated as a powerful tool for polymer crosslinking and preparation of materials with enhanced properties.

#### 3.3 Polymer surface modification

When modifying the surface of polymers, chemical selectivity typically plays a minor role, while harsh reaction conditions are useful for the modification of chemically inert substrates. Here, radical chemistry comes into play. When polymer surfaces are modified by radical chemistry, radicals are either generated directly on the polymers or on modifiers. In the latter case, a radical addition, substitution, or coupling reaction takes place to complete the modification. Radicals can be generated by a broader selection of homogeneous and heterogeneous approaches, including hydrogen atom abstraction, decomposition of immobilized initiators, electrochemical redox reaction, or irradiation because the reactions only need to take place at the surface.

Small-molecule oxidants, such as organic peroxides, hydrogen peroxide, persulfates undergo homolysis of O–O bonds generating radicals that can break C–H bonds followed by a hydrogen abstraction reaction. Phenolic compounds can be oxidized by molecular oxygen in the presence of laccase, and the resulting phenolic radical reacts with poly(ethersulfone) [143]. Highly reactive gaseous species may also generate radicals on polymer surfaces. For example, atomic oxygen radical anions emitted from 12CaO⋅7Al_2_O_3_ crystals [144] were used to modify PVC and polystyrene [145–146]. Plasma is also a powerful gas-phase tool for polymer surface modification and radical generation [147–148]. It can even generate radicals on otherwise inert fluoropolymer surfaces [149].

**Scheme 18:**
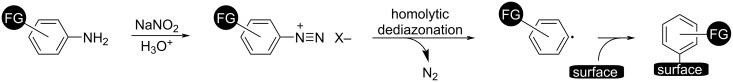
Polymer surface modification by homolytic dediazonation of diazonium salts.

Electrochemical reactions are another approach to generate radicals at polymer surfaces. Hydroxyl radicals generated via the electro-Fenton reaction from H_2_O_2_ in the presence of the Fe^3+^ were used to functionalize polypropylene surfaces [150–151]. Using a scanning electrochemical microscope, highly oxidative Ag(II) and NO_3_^·^ species were generated at a polymer surface [152], and oxidized the organic surface via a radical process. The homolytic dediazonation of diazonium salts produces highly reactive aryl radicals (Scheme 18) [153]. The chemical conversion can be initiated by electrochemical reduction [154], a reducing agent [155–157], a base [158], heating [159], or photochemically [160]. Aryl radicals may act as a halogen abstractor for alkyl halides and generate alkyl radicals for surface modification [161]. Electrochemical surface modification also works for inert PTFE surfaces in the presence of a 2,2’-dipyridyl redox mediator [162].

**Scheme 19:**
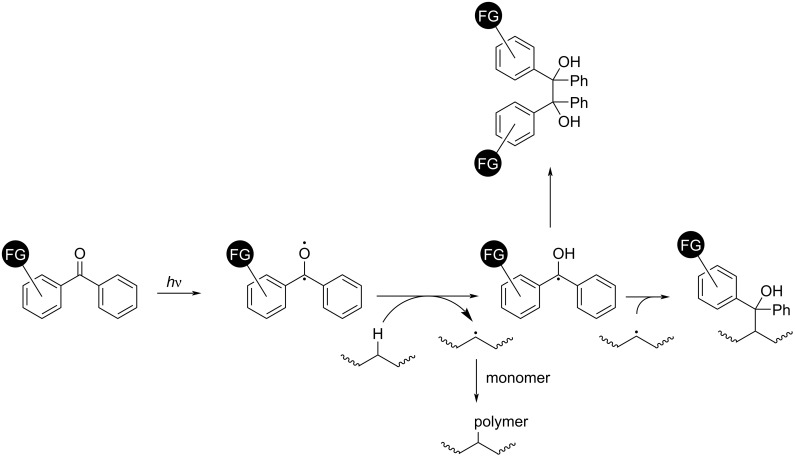
Photoinduced polymer surface modification or surface grafting using benzophenone.

Photons and high-energy charged particles can transfer their energy to bound electrons in atoms, exciting the electron to a higher energy level or even the vacuum, generating radical species. The energy of UV photons is comparable to the energy of chemical bonds [163], and therefore photons are particularly suitable for driving chemical reactions on polymer surfaces. Benzophenone is the best-established source of radicals on polymer surfaces. Its photoexcitation and subsequent reaction with polymers have been studied for decades [164–166]. When irradiated at around 360 nm, benzophenone undergoes excitation to a triplet state with biradical behavior. It then abstracts a hydrogen atom from the polymer resulting in a Ph_2_C^·^ species and a radical on the polymer (Scheme 19). This reaction may complete in radical coupling or proceed with radical polymerization from the surface [167–169], resulting in crosslinked polymers, surface-functionalized polymers, or surface-grafted polymers. RDRP was used to graft well-defined polymer brushes from polymer surfaces [170–171]. Photoinduced processes, including photoATRP and PET-RAFT were used [172–175]. Poly(aryl ether ketone)s such as poly(ether ether ketone), bearing a diaryl ketone moiety resembling that of benzophenone, can generate biradicals upon UV irradiation without a photoinitiator [176–177]. Grafted polymers and untethered polymers are generated simultaneously in the presence of a monomer.

Ionizing radiation including high-energy photons (X-rays and γ-rays) and charged α- or β-particles generate charged particles, especially electrons, emitted from the surface of polymers [178]. When a high-energy photon impacts an atomic electron, part of the photon energy is transferred to the electron leading to excitation or ionization and radical formation, and a deflected photon with lower energy is emitted, ready to impact another electron. This process is called Compton scattering [179]. One of the major purposes of radiation modification of polymer surfaces is grafting. The surface grafting can be simply tuned by the dose of radiation [180]. Radiation grafting on polymer surfaces is also compatible with RDRP for high density and well-defined polymer grafts [181–184]. Polymer surfaces can also be modified using electron and ion beams [185–186]. Komatsu et al. reported surface-initiated ATRP from electron-beam irradiated polymer surfaces [187].

The radical chemistry used for post-polymerization modification, crosslinking, and polymer surface modification has many aspects in common. The key is to activate chemically unreactive polymer backbones with highly reactive radical species to construct new chemical bonds.

### 4 Radical depolymerization

Radical destruction of polymer chains is an undesirable side reaction sometimes observed in the post-polymerization modification. However, it is also an important chemical process in several circumstances.

**Scheme 20:**
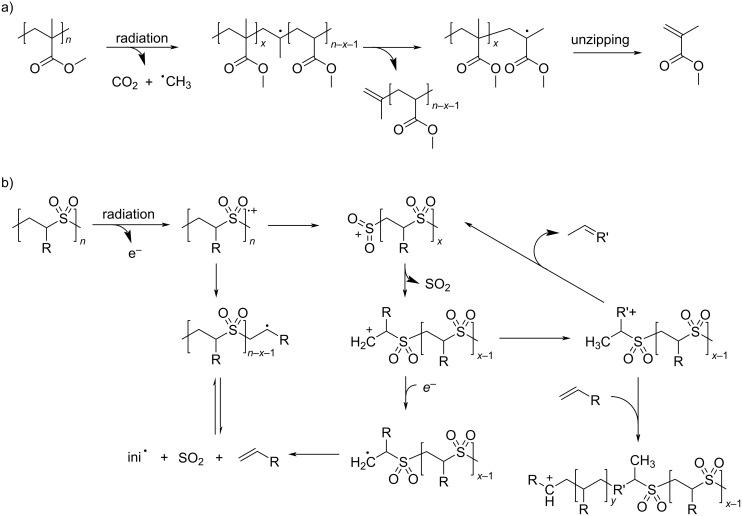
Depolymerization mechanism of common photoresists. (a) A possible mechanism of radiation decomposition of poly(methyl methacrylate). (b) A proposed mechanism of simultaneous radical/cationic decomposition of poly(olefin sulfone) upon radiation [197].

Photoresist is one of the bedrocks of the semiconductor industry [188–189]. There are two types of photoresists, positive and negative photoresists, and they become more or less soluble upon radiation, respectively. Some early negative photoresists undergo a photochemical crosslinking process of 1,3-diene cyclic polymers [190] (cf. section 3.2), but such systems are no longer studied due to the poor resolution and sensitivity. On the other hand, positive resists based on decomposition of polymers, especially upon radiation with an electron beam, because of its narrow wavelengths, are still regarded as a promising alternative. Poly(methyl methacrylate) has a long history of being used as a positive resist [191–192]. It undergoes a scission by a Norrish-type I reaction followed by radical unzipping depolymerization under photon or β-irradiation (Scheme 20a). Similarly, poly(olefin sulfone) undergoes depolymerization upon irradiation of light or electron beams [193–194]. It is an alternating copolymer of 1-olefins and SO_2_, and therefore the decomposition products are mostly gaseous [195]. While the depolymerization in both systems has a thermodynamic origin [196], the mechanism of poly(olefin sulfone) depolymerization is much more complex. Bowmer et al. proposed a simultaneous radical/cationic process (Scheme 20b) [197]. Meanwhile, an anionic process is also possible in the presence of a photogenerated base [198].

**Scheme 21:**
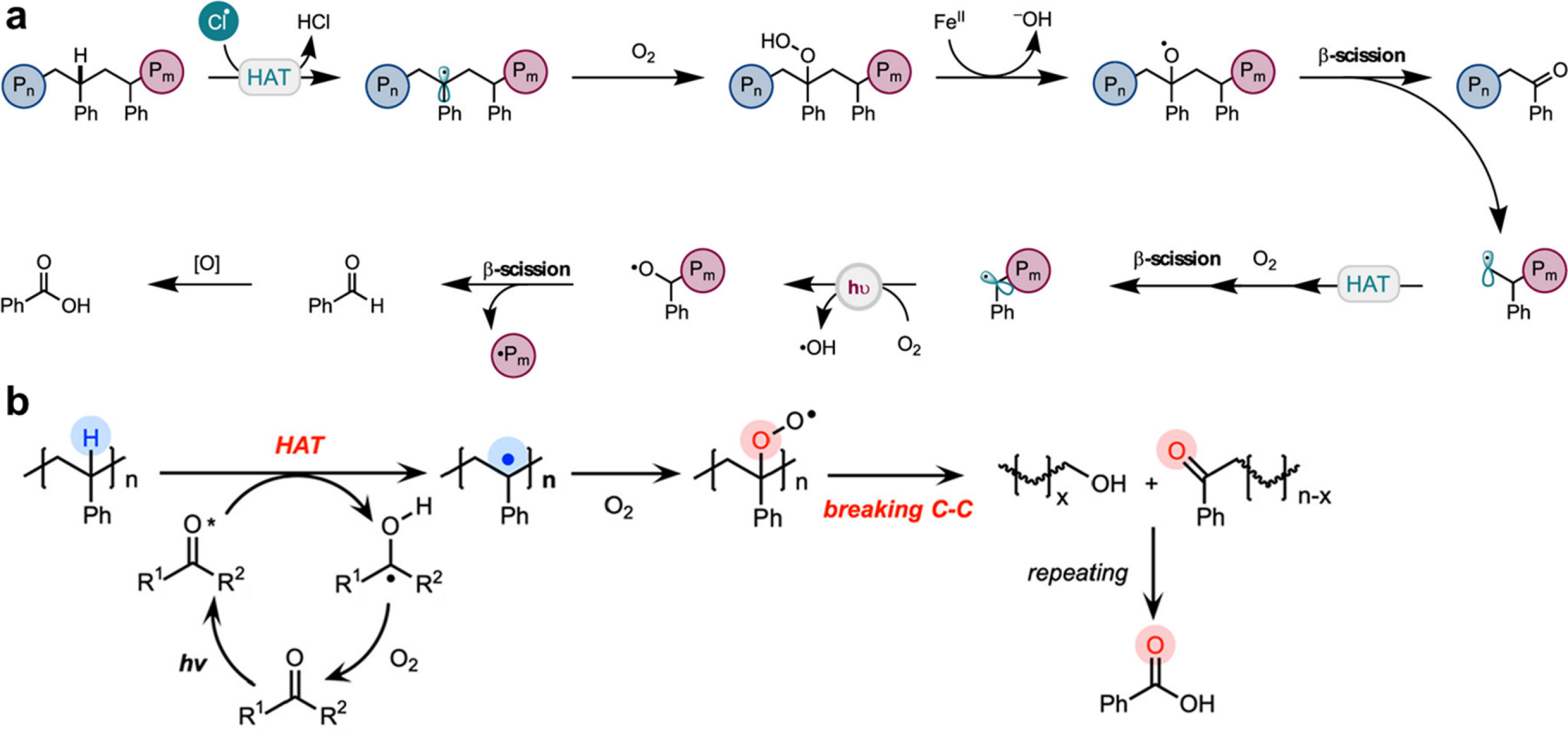
Proposed mechanisms of photooxidative depolymerization of polystyrene. (a) Scheme 21a was reprinted with permission from [202], Copyright 2022 American Chemical Society. This content is not subject to CC BY 4.0. (b) Scheme 21b was adapted from [203] (© 2022 T. Li et al., published by American Chemical Society, distributed under the terms of the Creative Commons Attribution 4.0 International License, https://creativecommons.org/licenses/by/4.0/ ).

Thanks to their low cost, light weight, and durability, polymeric materials are ubiquitous in modern life. However, over the past decades, people have become aware of the environmental impact of polymeric wastes [199]. One of the approaches to tackle this crisis is upcycling of polymeric wastes, i.e., chemical conversion of polymeric wastes into high-value raw materials [200]. Upcycling of polyesters has been extensively studied in recent years [201]. Nevertheless, upcycling of vinyl polymers, which comprise a major portion of commercial polymers, remains a great challenge because of their relatively unreactive backbones. Pyrolysis of such polymers is currently experimented by the industry to recover a variety of small molecules. Researchers have introduced radical depolymerization of vinyl polymers as a promising candidate for this task. Oh and Stache reported the photooxidation of polystyrene in the presence of FeCl_3_ as a radical source (Scheme 21a) [202]. A molar yield of 23% benzoyl small molecules was achieved. Reisner and co-workers employed a similar approach but using aromatic ketones as photocatalyst (Scheme 21b) [203]. Benzoic acid and other aromatic small molecules were recovered at a yield of ≈40% and ≈20%, respectively. Both processes were carried out under relatively mild conditions, paving a route toward a greener future of vinyl polymer upcycling. However, the yield and value of the small molecules produced in photooxidative depolymerization are still relatively low. Thermodynamics dominates the depolymerization of methacrylates [204–205]. Therefore, pyrolysis of PMMA gives a relatively high conversion to its monomer and the purification is straightforward [206–207].

Polyethylene and polypropylene make up a major fraction of commercial polymers. However, their upcycling is much more challenging. Uncontrolled radical depolymerization of these polymers in thermal processes typically gives low-value fuels and wax [208–210]. Kong et al. recently demonstrated a photothermal radical process for the conversion of polyethylene and polypropylene into blending compatibilizers [211]. Radical depolymerization capability can be incorporated at synthesis. Wang et al. introduced photodegradability to polyolefins by copolymerization of carbon monoxide [212]. Nevertheless, radical depolymerization is an essential tool to tackle the problem of polymer wastes.

## Conclusion

Radical chemistry has been deeply intertwined with the development of polymer science. Conventional free radical polymerization contributes to a major portion of modern polymer industry while novel polymerization techniques involving radicals emerged in the past decades to enable a rich selection of precisely controlled, high-value polymeric materials. The extremely high reactivity of radical species enabled efficient polymer modifications and depolymerizations with applications in many aspects essential to the advancement of the human society. Since the dawn of polymer science, it has been inextricably linked to organic chemistry. However, the two fields took divergent paths over the past century. Many emergent radical chemistries in the organic chemistry community has not yet found a place in the polymer science. We believe this gap will narrow with a broader use of chemical informatics tools and in-depth dialogs between the organic and polymer communities. Therefore, future opportunities for polymer science evolution lie in the collaboration of radical chemists in both communities.

Used abbreviations in the text and their explanations are collected in Table 2.

**Table 2:** Abbreviations used.

Abbreviation	Explanation

ATRA	atom transfer radical addition
ATRP	atom transfer radical polymerization
BCUP	di-*tert*-butylcumyl peroxide
BPO	benzoyl peroxide
CRP	controlled radical polymerization
CSIRO	Commonwealth Scientific and Industrial Research Organization
CTAs	chain transfer agents
DCP	dicumyl peroxide
DCPD	dicyclopentadiene
DOPA	ʟ-3,4-dihydroxyphenylalanine
HO-TEMPO	4-hydroxy-2,2,6,6-tetramethylpiperidine-1-oxyl
ITP	iodine transfer polymerization
IUPAC	International Union of Pure and Applied Chemistry
LDPE	low-density polyethylene
mfp	mussel foot protein
MMA	methyl methacrylate
MF-ROMP	metal-free ring opening metathesis polymerization
NMP	nitroxide-mediated polymerization
OMRP	organometallic-mediated radical polymerization
PAA	polyacrylic acid
PAN	polyacrylonitrile
PAM	polyacrylamide
PAT	poly(3-alkylthiophene)
PC	photocatalyst
PET-RAFT	photoinduced electron/energy transfer reversible addition–fragmentation chain transfer (polymerization)
PMMA	poly(methyl methacrylate)
PS	polystyrene
PTFE	polytetrafluoroethylene
PVA	poly(vinyl alcohol)
PVC	poly(vinyl chloride)
RAFT	reversible addition-fragmentation chain transfer
RDRP	reversible deactivation radical polymerization
RITP	reverse iodine transfer polymerization
ROMP	ring opening metathesis polymerization
TEMPO	2,2,6,6-tetramethylpiperidine-1-oxyl
UV	ultraviolet
XLPE	crosslinked polyethylene
